# FGF2 Alleviates Microvascular Ischemia-Reperfusion Injury by KLF2-mediated Ferroptosis Inhibition and Antioxidant Responses

**DOI:** 10.7150/ijbs.85692

**Published:** 2023-08-21

**Authors:** Fanfeng Chen, Jiayu Zhan, Mi Liu, Abdullah Al Mamun, Shanshan Huang, Yibing Tao, Jiaxin Zhao, Yu Zhang, Yitie Xu, Zili He, Shenghu Du, Wei Lu, Xiaokun Li, Zimiao Chen, Jian Xiao

**Affiliations:** 1 Department of Wound healing, The First Affiliated Hospital of Wenzhou Medical University, Wenzhou 325015, China.; 2 The Quzhou Affiliated Hospital of Wenzhou Medical University, Quzhou People's Hospital, Quzhou, China.; 3 Molecular Pharmacology Research Center, School of Pharmaceutical Science, Wenzhou Medical University, Wenzhou 325000, China.

**Keywords:** FGF2, Ferroptosis, Oxidative stress, Microvascular damage, Limb ischemia/reperfusion

## Abstract

An essential pathogenic element of acute limb ischemia/reperfusion (I/R) injury is microvascular dysfunction. The majority of studies indicates that fibroblast growth factor 2 (FGF2) exhibits protective properties in cases of acute I/R injury. Albeit its specific role in the context of acute limb I/R injury is yet unknown. An impressive post-reperfusion increase in FGF2 expression was seen in a mouse model of hind limb I/R, followed by a decline to baseline levels, suggesting a key role for FGF2 in limb survivability. FGF2 appeared to reduce I/R-induced hypoperfusion, tissue edema, skeletal muscle fiber injury, as well as microvascular endothelial cells (ECs) damage within the limb, according to assessments of limb vitality, Western blotting, and immunofluorescence results. The bioinformatics analysis of RNA-sequencing revealed that ferroptosis played a key role in FGF2-facilitated limb preservation. Pharmacological inhibition of NFE2L2 prevented ECs from being affected by FGF2's anti-oxidative and anti-ferroptosis activities. Additionally, silencing of kruppel-like factor 2 (KLF2) by interfering RNA eliminated the antioxidant and anti-ferroptosis effects of FGF2 on ECs. Further research revealed that the AMPK-HDAC5 signal pathway is the mechanism via which FGF2 regulates KLF2 activity. Data from luciferase assays demonstrated that overexpression of HDAC5 prevented KLF2 from becoming activated by FGF2. Collectively, FGF2 protects microvascular ECs from I/R injury by KLF2-mediated ferroptosis inhibition and antioxidant responses.

## Introduction

Acute limb ischemia is one of the most severe forms of peripheral artery disease, characterized by a sharp decline in limb blood flow, a high prevalence of amputations, irreversible limb necrosis and even death 1-3. Although ischemic tissue's quick reperfusion leads to better outcomes for patients, but there is no guarantee that microcirculatory blood flow will return. Postoperative complications as well as disease-related morbidity continue to occur at high rates 4. Therefore, new approaches to alleviate ischemia/reperfusion (I/R) limb injuries are highly warranted. Endothelial cell (EC) death is a common feature in I/R injury 5 as well as microvascular injury is the determinant of I/R injury in all tissues 6. Therefore, alleviating microvascular EC death is a key strategy for treating lower-limb I/R injury. Increasing evidence indicates that microvascular stress during I/R is exacerbated by impaired vascular function, thrombogenesis, increased interstitial fluid filtration, decreased capillary perfusion, leukocyte-endothelial cell adhesion, albumin seepage, as well as interstitial edoema 7-9. However, the precious molecular mechanisms of microvascular injury and EC death are still elusive. Alleviating EC death may provide a promising therapeutic strategy to treat and manage I/R-related diseases.

In the course of injury of I/R, oxidative stress (OS) is considered to be the primary initiator of EC death 6. OS signals are triggered by an increase in reactive oxygen species (ROS) production, which disrupt the microvascular barrier and increase vascular permeability, the release of inflammatory mediators and apoptotic factors 10. Ferroptosis, a programmed cell death distinct from apoptosis, necrosis and autophagy, is characterized by lipid peroxidation 11. lipid peroxidation further promotes ROS generation, leading to the activation of ferroptosis-dependent cell death. In addition, iron ions can enhance the generation of ROS through the Fenton reaction, thereby aggravating OS 12. Ferroptosis is associated with several physiological and pathological processes. Current evidence indicates that ferroptosis is closely implicated in I/R injury in the heart, brain, liver and kidney 11, 13. It is still unknown how ferroptosis affects I/R injury in lower-limb I/R injury. Therefore, we postulated that ferroptosis contributes to EC death in acute limb I/R injury.

The Kruppel-like factor (KLF) family of transcriptional regulatory proteins regulates various gene expressions through transcriptional regulation 14. KLF2 is a crucial family member, inhibits vascular calcification, maintains vascular integrity and promotes angiogenesis 15-17. Current researches have revealed that KLF2 protects EC functions by mediating laminar flow and enhancing anti-oxidant activity 18, 19. Pioneering evidence suggests that KLF2 alleviates thrombosis, inhibits OS and mitigates inflammation in models of I/R injury 20, 21. Wu et al. report that KLF2 can regulate endothelial nitric oxide synthase in ECs via nuclear factor E2-related factor 2/heme oxygenase-1 (NFE2L2/HO-1) under hypoxia as well as reoxygenation conditions and protects EC function 22. NFELE2 is a key regulatory factor for cells to maintain oxidative stability. By attaching to the anti-oxidant response elements (AREs) in the nucleus, the transcription of target genes and the translation of anti-oxidant and anti-inflammatory proteins can be encouraged by NFELE2, thereby promoting cell protection 23. Moreover, it has been demonstrated that KLF2 obstructs oxidation reactions by activating NFE2L2/ARE signaling *in vivo* and *in vitro* and exerts anti-oxidative functions 24. Earlier evidence reports that NFELE2 activates the expression of multiple target genes that regulate ferroptosis by controlling glutathione (GSH), iron, lipid metabolism and mitochondrial activity 25. Dong and co-workers have recently suggested that NFELE2 suppresses ferroptosis by upregulating the expression of HO-1 as well as solute carrier family 7, member 11 protein (SLC7A11) 26. The SLC7A11 (also known as xCT) gene belongs to the solute transporter family and encodes cystine/glutamate reverse transporter, an essential gene that regulates ferroptosis 27. Glutathione peroxidase 4 (GPX4), downstream of SLC7A11, is a central regulator of ferroptosis inhibition via changing harmful lipid alcohols from lipid hydroperoxides 28. Current evidence indicated that NFELE2 inhibits ferroptosis and alleviates I/R injury by regulating the SLC7A11/GPX4 signaling pathway 26.

Within the fibroblast growth factor (FGF) family, FGF2 is an essential component. FGF2 modulates a variety of cellular processes by binding and activating FGF receptor 29. Previous studies have revealed that FGF2 promotes angiogenesis, wound healing and tissue regeneration 30, 31. FGF2 inhibits OS and mitigates inflammation 32, 33, reduces myocardial infarct size and ameliorates myocardial dysfunction 34, 35. Adenosine-monophosphate-activated protein kinase (AMPK) is a serine/threonine protein kinase complex. Accumulating evidence shows that AMPK plays a vital role in recovering limbs from I/R-associated diseases 36, 37. There is strong evidence suggest that FGF2 increases AMPK phosphorylation, which starts the AMPK signalling cascade 38. Histone deacetylase 5 (HDAC5) is one of the classes IIa HDACs whose activity is regulated by its phosphorylation-dependent nuclear/cytoplasmic shuttling 39. More importantly, AMPK pathway activation further promotes the HDAC5 translocation from the nucleus to the cytoplasm 40. Emerging evidence indicates that inhibition of KLF2 activates NFELE2 41, 42. Therefore, our research concentrated on determining whether FGF2 can ameliorate microvascular I/R injury by restricting OS and suppressing ferroptosis-mediated cell death.

## Materials and Methods

The Institutional Animal Care & Research Ethics Committee from Wenzhou Medical University (wydw 2017-096) approved the study's animal experimental methods and procedures, which adhered to the China National Institutes of Health's Guide for the Care and Use of Laboratory Animals. Healthy, adult male C57BL/6 mice (age = around 6 weeks and average weight: 18-23g) were offered by Wenzhou Medical University (Licence no. SCXK 2005-0019) and were kept under normal conditions (temperature of 21-25 °C, relative humidity: 45-55%, 12-h light/dark period) and unrestricted access to water and food.

### Antibodies and reagents

The following chemicals were employed in our research: FGF2 was supplied from the biotechnology pharmaceutical engineering lab at Wenzhou Medical University and synthesized based on previous work. Masson Staining Kit and BCA Kit are both available from Thermo Fisher Scientific in the United States and Solarbio Science in Beijing, China, respectively. The following companies provided the specific primary antibodies that were needed: HO-1 (10701-1-AP), NQO1 (11451-1-AP), SOD1 (24127-1-AP), AMPK (66536-1-lg), NFE2L2 (16396-1-AP), GAPDH (60004-1-lg) and Histone-H3 (17168-1-AP) from Proteintech Group; SLC7A11 (DF12509) and KLF2 (DF13602) from Affinity; GPX4 (381958) from ZEN Bio; p-AMPK (2535) from abcam; HDAC5 (A0632) and p-HDAC5 (AP0202) from Abclonal; 4-HNE (MAB3249-SP) from R&D Systems. CD31 (sc-376764) antibody from Santa Cruz. The secondary IgG antibody coupled with horseradish peroxidase (HRP) was purchased from Santa (Cruz, CA, USA).

### Model of acute hind limb I/R injury

An orthodontic rubber band was placed over the greater trochanter of the right leg in order to construct the I/R injury model 43. The rubber band was removed after 4 hours of ischemia, and 24 hours of reperfusion followed. Similar procedures were followed by the sham group, with the exception of applying rubber bands.

### Drug administration

I/R groups were distributed into I/R+FGF2 group, I/R+Ferrostatin-1 (Fer-1) group, I/R+FGF2+Erastin group, I/R+Erastin group, I/R+FGF2+ML385 group, I/R+ML385 group, I/R+FGF+LV-KLF2 group, I/R+FGF2+LV-Con group, I/R+LV-KLF2 group, I/R+FGF2+Compound C (CC) group and I/R+CC group. FGF2 group received injections of FGF2 (2 mg/kg, i.p.) at three-time points: right before ischemia, before reperfusion and 30 min post-reperfusion. Two weeks previous to the surgery, lentivirus KLF2 was intramuscularly injected into the right gastrocnemius muscle for both the I/R+LV-KLF2 and I/R+FGF2+LV-KLF2 groups. Equivalent quantities of lentivirus vehicles encoding the negative control sequence were administered to the control group. Three days before to surgery, intraperitoneal injections of Fer-1 (5 mg/kg, MCE, HY-100579) and Erastin (20 mg/kg, MCE, HY-15763) were given daily to the I/R+Fer-1 group, I/R+Erastin group and I/R+FGF2+Erastin group. Injections of ML385 (30 mg/kg, i.p., MCE, HY-100523) were performed in the I/R+ML385 and I/R+FGF2+ML385 groups with the same protocol. Three days prior to surgery, CC (10 mg/kg, MCE, HY-13290) intraperitoneal injections were administered daily to the I/R+CC as well as I/R+FGF2+CC group and I/R+FGF2+CC group.

### Laser doppler imaging (LDI)

Under a state of general anaesthesia, LDI was performed on each mouse.The LDI, a laser doppler device with significant penetration, made small vessels visible at great depth and helped to clarify the blood flow to the hind limb 44. Then, the hind limb of the tranquillized mice was subsequently subjected to Laserflo BPM scanning (Moor Instruments, UK). Blood flow rates are shown via perfusion imagery analysis, ranging from low (blue) to high (red). The flow rate was determined using Application called Moor LDI Review (version 6.1; Moor Instrumentation) as well as expressed in perfusion units (PU). Each animal underwent three scans, and the mean of those readings was derived prior to group comparison.

### Skeletal muscle edoema assessment

Skeletal muscles were harvested after reperfusion. The muscle wet-to-dry weight ratio was determined to analyze muscle edema. Skeletal muscles were obtained post-reperfusion, and the proportion of wet to dry weight was computed to assess muscular edoema. Tissue samples were taken, weighed right away, and then dried at 55 °C till a consistent weight was obtained.

### Masson staining

After perfusion, gastrocnemius muscle tissues were obtained. The muscle samples were paraffin-embedded, formalin-fixed, and sectioned at a thickness of 5-μm. Tissue sections treated with PFA and enclosed in paraffin received appropriate dewaxing and dehydration. Following the guidelines provided by the manufacturer, a kit was used to carry out the Masson's trichrome staining procedure. Based on their shape, muscle fibres were then classified as either undamaged or damaged. A proportion of damaged fibres for each leg was used to display the final statistics 45.

### Cell culture and treatments

The School of Pharmaceutical Science (Wenzhou Medical University, Zhejiang Province, China) provided the human umbilical vein endothelial cells (HUVECs) for this study. HUVECs were cautiously cultivated in DMEM added with FBS (10%, fetal bovine serum), penicillin (100 U/mL) and kyowamycin (100 μg/mL) under 37 °C in a humidified cell incubating device with continuous 5% CO2 supply.

During the cells' logarithmic development phase, the oxygen-glucose deprivation/re-oxygenation (OGD/R) model was developed. Initial media replacement involved switching to serum-free RPMI 1640 medium from GIBICO, and cells were then put into a three-gas incubator from Thermo Scientific that contained 95% N_2_ and 5% CO_2_, lowering oxygen levels to 1%. Cells were cultured for 8 hours, and then they were cultivated for 16 hours in a typical DMEM high-glucose medium. Prior to hypoxia, all prescribed treatments were given: lentivirus KLF2 for 48 hours and FGF2 (20 ng/mL) during 24 hours.

Cell viability was measured using CCK8 (Beyotime, Shanghai, China) reagent following manufacturer's instructions. To measure cell viability, the OD450 value was normalised to the control group.

### HUVEC Immunofluorescence staining

HUVECs were grown on well pate with coverslips overnight. They were adequately blocked with 5% bovine serum albumin (BSA) at room temperature (RT) for 30 minutes after being fixated with 4% paraformaldehyde (PFA) for 15 minutes. After that, NFE2L2, KLF2, and GPX4-specific primary antibodies were incubated on the coverslips for an entire night at 4 °C. Cells were carefully cleaned 3 times via PBS for five minutes each. The cells were then incubated for 1 h at room temperature with fluorescence-linked secondary antibodies. After that, PBS was used to rinse the coverslips to get rid of the secondary antibodies. Before mounting the glass plate with the DAPI Fluoromount-GTM (Yeason, 36308ES20) reagent, any PBS leftovers were cleaned off of it. The Nikon ECLIPSE 80i microscope (Nikon, Japan) was used to take and analyse digital pictures.

### Measurement of ROS production *in vitro*

The DCFH-DA, an oxidation-sensitive fluorescent probe was employed to suspend pure HUVECs and HUVECs were incubated *in vitro* for 20 minutes at the typical 37 °C. Using a confocal microscope (Nikon, Japan), cell fluorescence was captured after sufficient washing with serum-free media. Intracellular ROS was measured with the ROS assay kit (Beyotime, S0033) in accordance with the manufacturer's instructions.

ROS generation was calculated *in vivo* using DHE staining. Fresh skeletal muscle samples were cut into cryosections and treated with DHE (5 mol/L, Sigma Aldrich, D7008) for 30 minutes at 37 °C. The samples were then analysed using a confocal microscope (made by Nikon, Japan).

### Western blotting

Muscle and cell samples were homogenized in ice-cold RIPA buffer (Solarbio, R0010) containing protease inhibitor cocktail (Solarbio, R0010) and phosphatase inhibitor cocktail (Abcam, GR304037-28). Following the directions provided by the manufacturer, a BCA protein assay kit was used to measure the protein concentrations. On polyvinylidene difluoride membranes, protein samples were separated utilising 12.5% SDS-PAGE. After sufficiently blocking with 10% fat-free milk solution, the specified primary antibodies were added to the membranes and incubated overnight at 4 °C: HO-1, NQO1, SOD1, NFE2L2, GAPDH, GPX4, SLC7A11, Histone H3, AMPK, p-AMPK, HDAC5, p-HDAC5, KLF2, FGF2 as well as 4-HNE. After that, the membrane was exposed to HRP-conjugated IgG secondary antibody for 1.5 hours at room temperature. The bands have been observed and quantified employing Bio-Rad's Image Lab 3.0 software.

### GSH, Malondialdehyde (MDA) and ferrous ion (Fe^2+^) assays

The Total Glutathione detection kit (Assay Designs) was employed to measure the levels of GSH in both cells as well as muscle tissue while following the manufacturer's instructions. With measures made at 532 nm, the colorimetric assay mainly determined MDA concentrations in cells as well as muscle tissue. Employing the Iron Assay Kit (Abcam), iron ion concentrations inside cell as well as muscle tissue were measured.

### JC-1 staining

JC-1 dye (Beyotime, C2006), a carbocyanine dye with cationic fluorescence characteristics, was used as a ratiometric indicator to measure mitochondrial membrane potential (MMP) in cells. Cells underwent two washes with the JC-1 buffer solution after being incubated with the JC-1 dye solution (2.5 g/mL) for 20 minutes in the dark at 37 °C. A fluorescent microscope and an argon-ion 488 nm laser were used to determine MMP by measuring the proportions of mitochondrial JC-1 monomers or aggregates. Healthy cells with aggregated JC-1 emitted red fluorescence, whereas injured cells with reduced MMP showed green fluorescence caused by monomeric JC-1. MMP was quantified using the ratio of red-to-green fluorescence.

### Transcriptome sequencing

The genome-wide transcriptomics analyses were carried via (LC-BIOTECHNOLOGIS (HANGZHOU) CO., LTD) after total RNA was extracted. Using the R software edger or DESeq2, the differently expressed mRNAs were screened with fold change (FC) greater than 2 or FC less than 0.5 and p-value less than 0.05. Differentially expressed genes (DGEs) were grouped hierarchically to reveal patterns of gene expression in various populations. On the basis of hypergeometric distribution, an independent GO enrichment analysis of DEGs was carried out using R.

### Luciferase assay

To investigate the putative regulatory function of FGF2 within KLF2 transcriptional activity, a luciferase reporter assay was carried out. The transcriptional activity of KLF2 was assessed by the KLF2-driven luciferase activities. Based on the manufacturer's instructions, HEK293T cells were co-transfected with pRL-TK, pGL3-basic, or pGL3-KLF2 and empty pcDNA3.1 vector or HDAC5-pcDNA3.1 plasmids in 24-well plates using liposome transfection reagent 2000 (Invitrogen, Carlsbad, CA). Cells were transfected for 24 hours before receiving 20 ng/ml of FGF2. 48 hours after transfection, Firefly and Renilla luciferase activities were both detected using the Dual-Luciferase® Reporter Assay System (Promega) as well as a microplate reader (Synergy H1, Bio-Tek) to determine the firefly/Renilla luciferase ratio.

### Statistical analysis

Software from SPSS, version 19, was used to analyse the data (Chicago, IL, USA). The mean ± standard deviation (SD) for each experimental data point were presented. To detect group differences, independent-specimen t tests were employed. To identify differences between multiple groups, ANOVA with LSD (equal variances assumed) or Danett T3 (equal variances not assumed) multiple comparison (post hoc) tests were used to evaluate statistical differences. A two-tailed p-value of less than 0.05 was regarded as statistically significant.

## Results

### FGF2 expression increases after acute limb I/R injury

**Figure 1:**
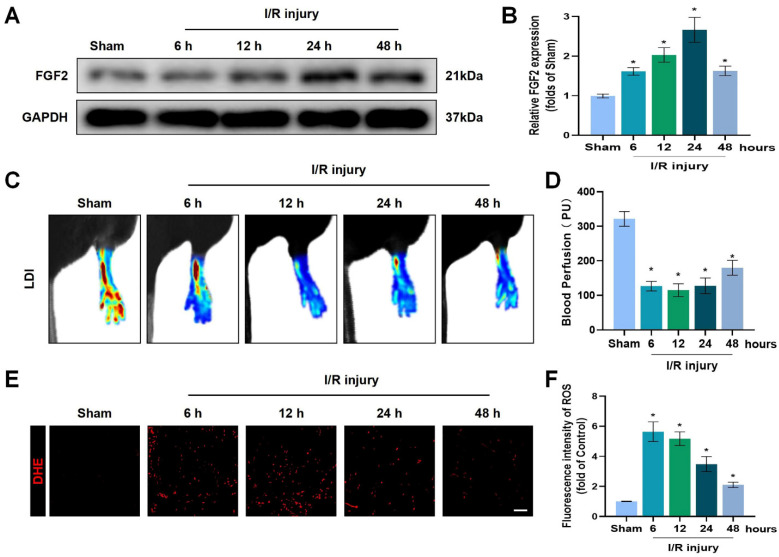
Increased FGF2 expression after acute limb I/R injury. (**A**) WB evaluation of skeletal muscle samples for FGF2. (**B**) Quantification of the FGF2 protein level from (A), standardised to GAPDH band density. (**C**) LDI analysis of post-I/R hind limb blood perfusion. (**D**) A histogram showing the strength of the blood flow signal. (**E**) DHE staining used to measure the ROS levels in limbs damaged by I/R. (**F**) A histogram displaying the signal strength of ROS levels. Data are presented as mean ± SD, (n = 3-5 per group). Significance: ^*^P < 0.05.

To explore the pathophysiological significance of FGF2 after I/R injury, the gastrocnemius muscles of limbs were excised from multiple time points of reperfusion and FGF2 levels were assessed. A western blot assay revealed that FGF2 levels significantly increased after I/R damage, as measured at different stages of reperfusion. After then, FGF2 levels stabilised (Fig. 1A, B). After I/R, there existed a significant drop within blood flow, which persisted for up to 48 hours compared to the sham group (Fig. 1C, D). DHE staining was used to measure the ROS levels, which augmented and then felled after I/R injury (Fig. 1E, F). These results imply that FGF2 has a protective role in the limbs after an acute I/R damage.

### FGF2 reduces limb tissue edoema and skeletal muscle fibre damage in limbs following I/R injury and improves blood perfusion and microvascular density

**Figure 2:**
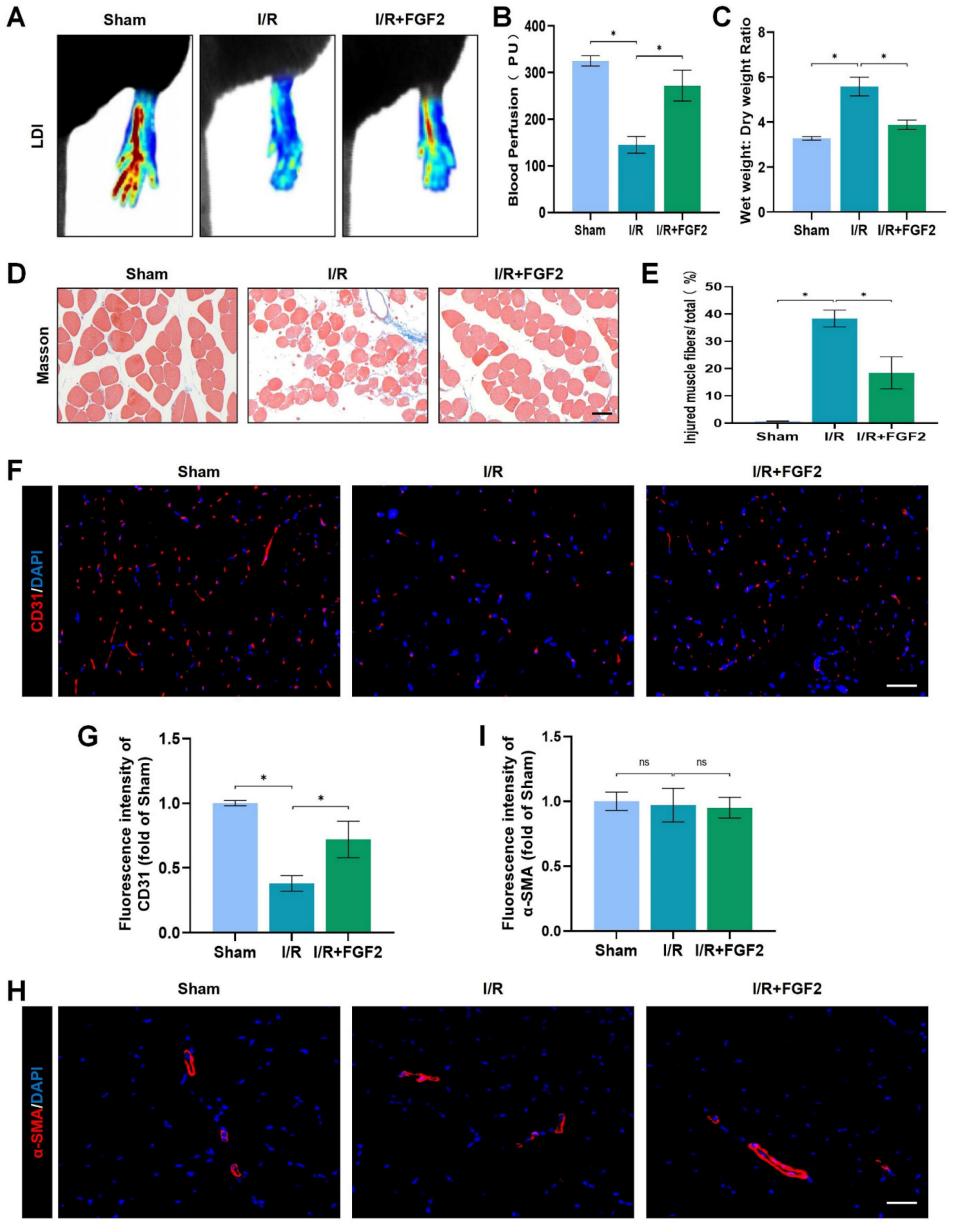
FGF2 reduces limb tissue edema and skeletal muscle fibres injury while increasing blood perfusion and microvascular EC density in I/R limbs. **(A)** LDI analysis of the blood perfusion in the hind limbs. (**B**) A histogram showing the strength of the blood flow signal. (**C**) Wet weight to dry weight ratio. (**D**) Transverse slices of skeletal muscle stained with Masson. Scale bar: 100 µm. (**E**) Calculating the fraction of damaged fibres in skeletal muscle. (**F**) Images of skeletal muscle sections labelled with CD31 antibodies and showing DAPI staining to identify the nuclei. Scale bar: 100 µm. Using the immunofluorescence data in (F), the average CD31 optical density is shown in **(G)**. (**H**) Images of skeletal muscle sections stained with anti-α-SMA antibodies and showing DAPI-positive nuclei. Scale bar: 100 µm. Immunofluorescence data in (H) were used to calculate the average α-SMA optical density in **(I)**. Data are presented as mean ± SD (n = 3-5 per group). Significance: ns, not significant; ^*^P < 0.05.

In mice subsequent I/R damage, the possible therapeutic role regarding FGF2 was investigated. When compared to the sham group, the I/R group had severe limb hypoperfusion, according to LDI's initial assessment of limb blood perfusion. Contrarily, the FGF2 group displayed more strong signs of muscle blood flow compared to the I/R group, suggesting that FGF2 plays a substantial role in improving limb blood perfusion following I/R injury (Fig. 2A, B). By lowering the wet-to-dry weight ratio, FGF2 was also seen to lessen skeletal muscle edoema (Fig. 2C). We also assessed the degree of muscle fibre damage, which was expressed as a percentage (%) of injured fibres. An important finding was that the I/R group had severe muscle fibre injury, which was characterised by cellular wall/membrane disruption, cytoplasmic alterations, and loss of polygonal cell shape and cell adhesion. Quantitative analysis revealed that the FGF2-treated cohorts had less fibre damage than the I/R injury group (Fig. 2D, E). Additionally, the effect of FGF2 on microvascular density during I/R damage was evaluated. The results showed a marked decrease in microvascular density, which was supported by weaker CD31 signals in the skeletal muscle after I/R (Fig. 2F, G). While -SMA (vascular smooth muscle marker) signals were unaltered by I/R damage or FGF2 treatment, CD31 signals were elevated by FGF2 intervention (Fig. 2H, I). According to these findings, FGF2 effectively counteracts I/R-induced hypoperfusion, reducing skeletal muscle and microvascular damage.

### FGF2 mitigates I/R-induced OS in ECs

**Figure 3:**
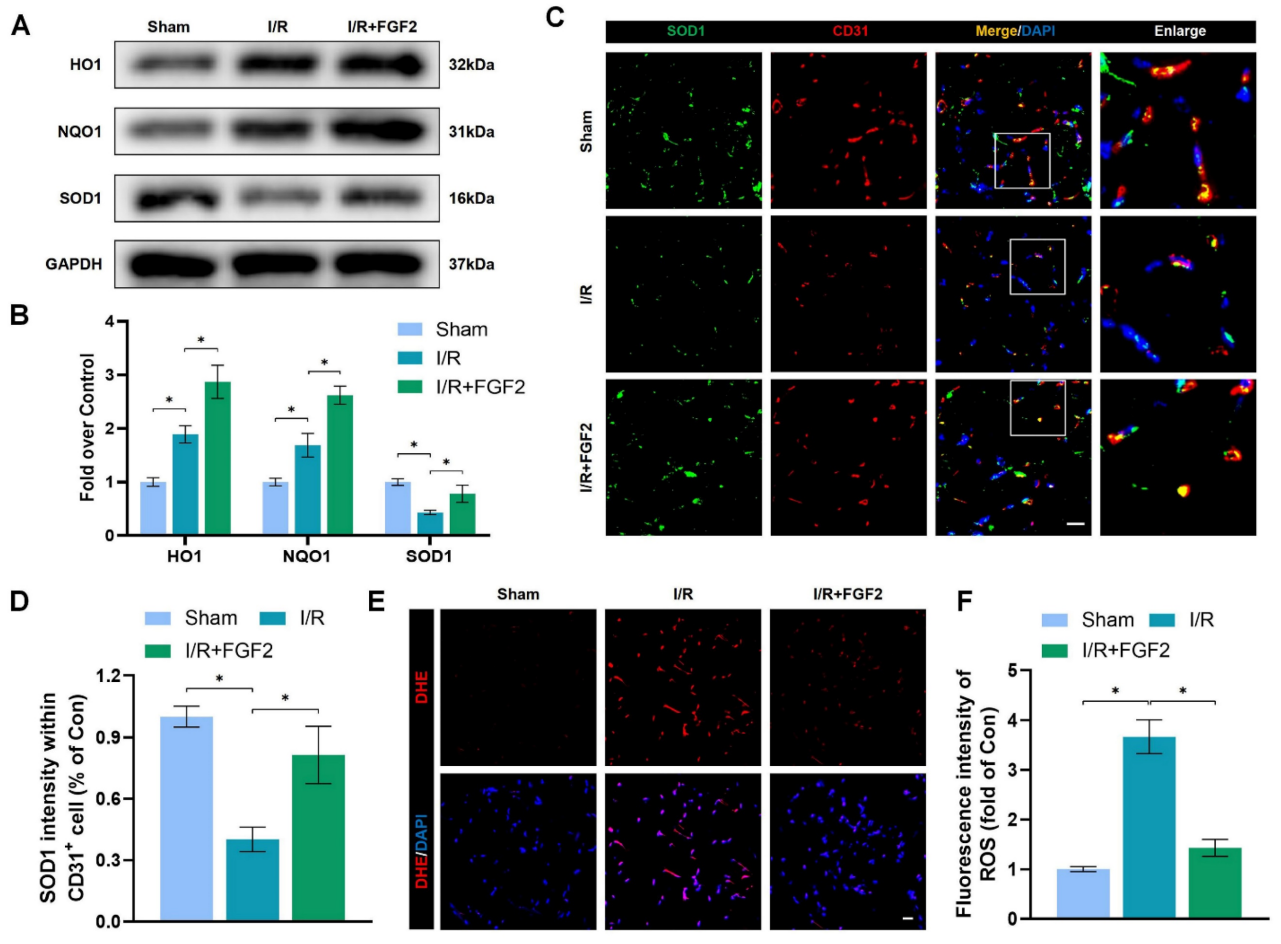
FGF2 reduces I/R-induced oxidative stress in ECs. (**A**) WB assay of skeletal muscle samples with HO-1, NQO1, and SOD1. (**B**) Quantification of HO-1, NQO1, and SOD1 proteins at the protein level from (A), normalised to GAPDH band density. (**C**) Skeletal muscle slices with SOD1 and CD31, an EC marker, stained. DAPI staining is used to identify the nuclei. Scale bars: 100 μm. (**D**) Quantification of SOD1 and CD31 cells with double positivity and percentages of total CD31 positive cells. (**E**) Images of skeletal muscle sections stained with DHE, nuclei were recognized by DAPI staining. Scale bars: 100 μm. Using the immunofluorescence data in (E), the average ROS optical density is shown in **(F)**. Data are presented as mean ± SD (n = 3-5 per group). Significance: ^*^P < 0.05.

OS and cellular damage are crucial to I/R injury aetiology. We examined the effect of FGF2 on oxidative stress *in vivo*. Using a western blot method, we evaluated the expression of proteins linked to OS. More importantly, FGF2 treatment substantially raised the protein expression of HO-1, NQO1, and SOD1 in skeletal muscle after I/R damage (Fig. 3A, B). Strangely, skeletal muscle after I/R injury showed less SOD1 fluorescence intensity on the vascular wall than control groups (Fig. 3C, D). Following the treatment of FGF2, increased SOD1 fluorescence intensity on the vascular wall was observed. ROS levels were determined using DHE staining (Fig. 3E, F), which demonstrated that FGF2 effectively reduced ROS levels caused by I/R. These findings imply that FGF2 is essential for reducing OS levels in ECs after I/R injury.

### FGF2 prevents I/R-induced ferroptosis in ECs

**Figure 4:**
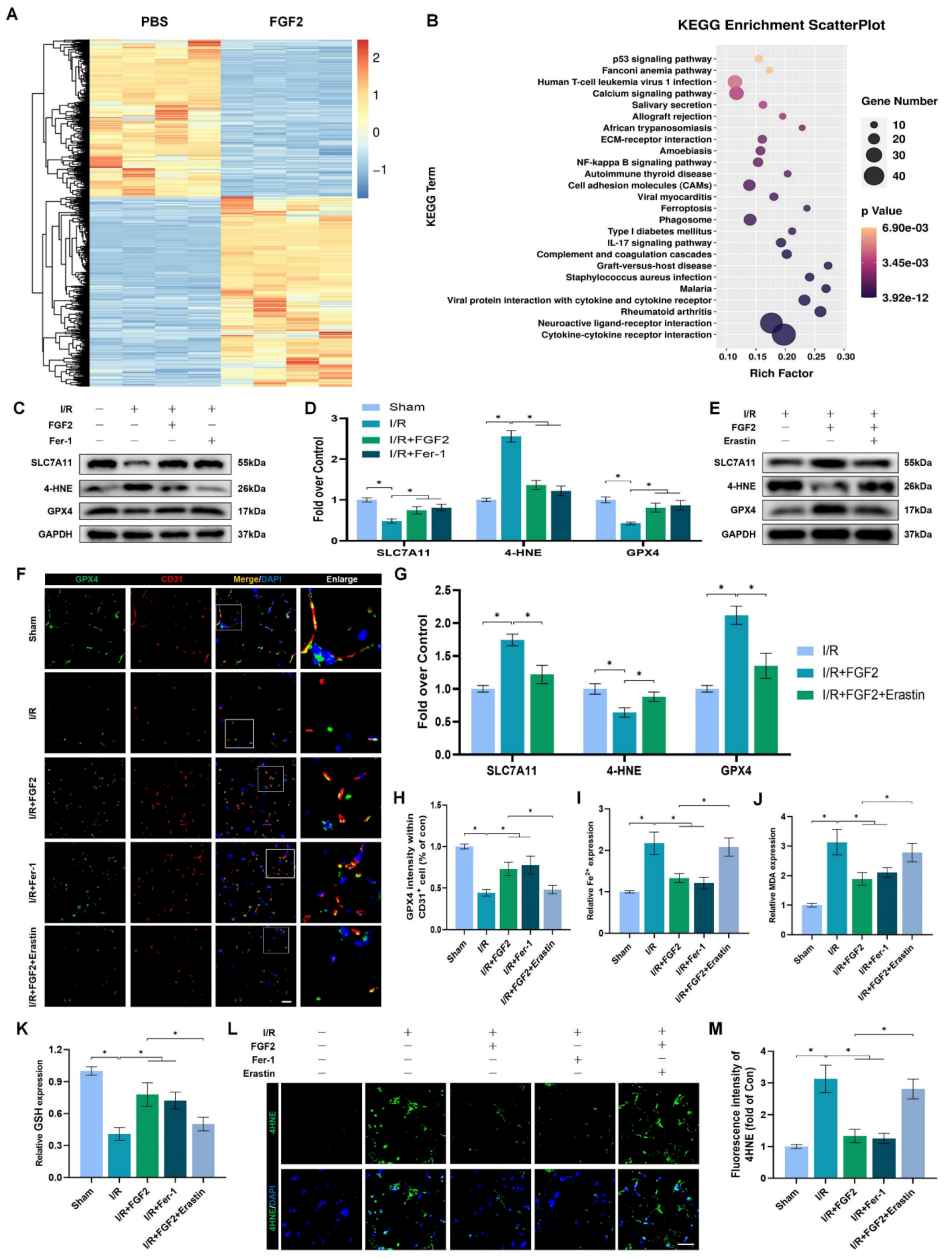
FGF2 reduces I/R-induced ferroptosis in ECs. (**A**) Heatmap displays the PBS and I/R cohorts' distinctive abundance characteristics. (**B**) The KEGG database's functional classification of the FGF2-regulated genes. (**C**) WB assay of GPX4, 4-HNE, and SLC7A11 in skeletal muscle tissues. (**D**) Quantification of protein level of SLC7A11, 4-HNE and GPX4 from (C) with normalised to GAPDH band density. (**E**) WB assay of GPX4, 4-HNE, and SLC7A11 in skeletal muscle samples. (**F**) Skeletal muscle slices with GPX4 and CD31, an EC marker, stained. DAPI staining is used to identify the nuclei. Scale bars: 100 μm. (**G**) Quantification of protein level of SLC7A11, 4-HNE and GPX4 from (E) with normalized with respect to GAPDH band density. (**H**) Quantification of GPX4 and CD31 double-positive cells, the percentages of double positive cells versus total CD31 positive cells are indicated. (**I**) Content of Fe^2+^ was plotted as a histogram. (**J**) A histogram displaying the MDA content. (**K**) Content of GSH was plotted as a histogram. (**L**) Sections of skeletal muscle stained with 4-HNE, with DAPI staining identifying the nuclei. Scale bars: 100 μm. Based on the immunofluorescence results in (L), the average 4-HNE optical density is shown in **(M)**. Data are presented as mean ± SD (n = 3-5 per group). Significance: ^*^P < 0.05.

We investigated the molecular mechanisms behind FGF2's protective effects on limb I/R injury. With 967 upregulated and 791 downregulated genes, there were 1758 DEGs when the transcriptomes of the PBS and FGF2-treated cohorts were compared. KEGG analysis revealed that, compared with other types of cell death, these DEGs were mainly predominant in cell ferroptosis (Fig. 4A, B). Considering that excessive oxidative stress is often accompanied by excessive accumulation of ROS and malondialdehyde (MDA) (the characteristic indicators related to ferroptosis) 12 and the potent antioxidant effects of FGF2 mentioned above, we hypothesized that FGF2 protects limb I/R injury by suppressing ferroptosis.

The examination of ferroptosis-associated protein expression in ECs following I/R injury is made possible by the use of WB. Fer-1, a ferroptosis inhibitor, was included as a positive control. Importantly, our WB analysis revealed that FGF2 treatment significantly increased SLC7A11 and GPX4 protein expression degrees within the skeletal muscle tissue after I/R impairment. In addition, FGF2 dramatically decreased the levels of 4-Hydroxynonenal (4-HNE) protein expression in skeletal muscle tissue following I/R damage (Fig. 4C, D). The above changes in protein level induced by FGF2 were comparable to that in the Fer-1 group (Fig. 4C, D).

Next, we used Erastin, an agonist of ferroptosis and found that Erastin significantly reduced GPX4 protein levels in the I/R group (Fig. S1F, G). Besides, enhanced SLC7A11 and GPX4 and decreased 4-HNE induced by FGF2 were counteracted by Erastin application (Fig. 4E, G). Immunofluorescence staining indicated that FGF2 enhanced the GPX4 fluorescence intensities on the vascular wall in skeletal muscle. Intriguingly, our results also revealed that GPX4 fluorescence intensity was abrogated by Erastin (Fig. 4F, H). We found that FGF2 intervention markedly minimized 4-HNE fluorescence intensity within skeletal muscle tissue after I/R injury. However, the reduced fluorescence intensity of 4-HNE was increased by the administration of Erastin (Fig. 4L, M).

We also detected other indicators of ferroptosis: MDA is a product of membrane lipid peroxide, which represents positively correlated with ferroptosis; Iron ions are the key to ferroptosis and detecting the content of iron ions can indicate whether ferroptosis occurs; reduced GSH is inversely associated with ferroptosis. Our data showed that the content of MDA were observed to be higher in the I/R group compared to the sham group, associated with higher iron ion levels and lower GSH levels. While, enhanced anti-ferroptosis effect of FGF2 was abolished by the Erastin application (Fig. 4I-K).

We then verified FGF2's potential contribution to limb I/R injury defence via regulating ferroptosis. FGF2 administration significantly enhanced limb perfusion and was comparable to those treated with the Fer-1. Intriguingly, increased blood perfusion was reversed by Erastin administration (Fig. S1A, B). In addition, decreased wet-to-dry weight ratio induced by FGF2 indicated that FGF2 ameliorated muscle swelling after I/R injury, which was comparable to the Fer-1-treated group. This protective role was also reversed by the Erastin administration (Fig. S1C). Furthermore, FGF2 intervention could alleviate muscle fiber injury, comparable to the Fer-1-treated group. We found that the reduced injured muscle fiber resulting from FGF2 was reversed by Erastin (Fig. S1D, E). Collectively, these findings point to FGF2's protective function against limb I/R injury, perhaps by preventing EC ferroptosis.

### FGF2 inhibits OS and ferroptosis by activating NFE2L2

**Figure 5:**
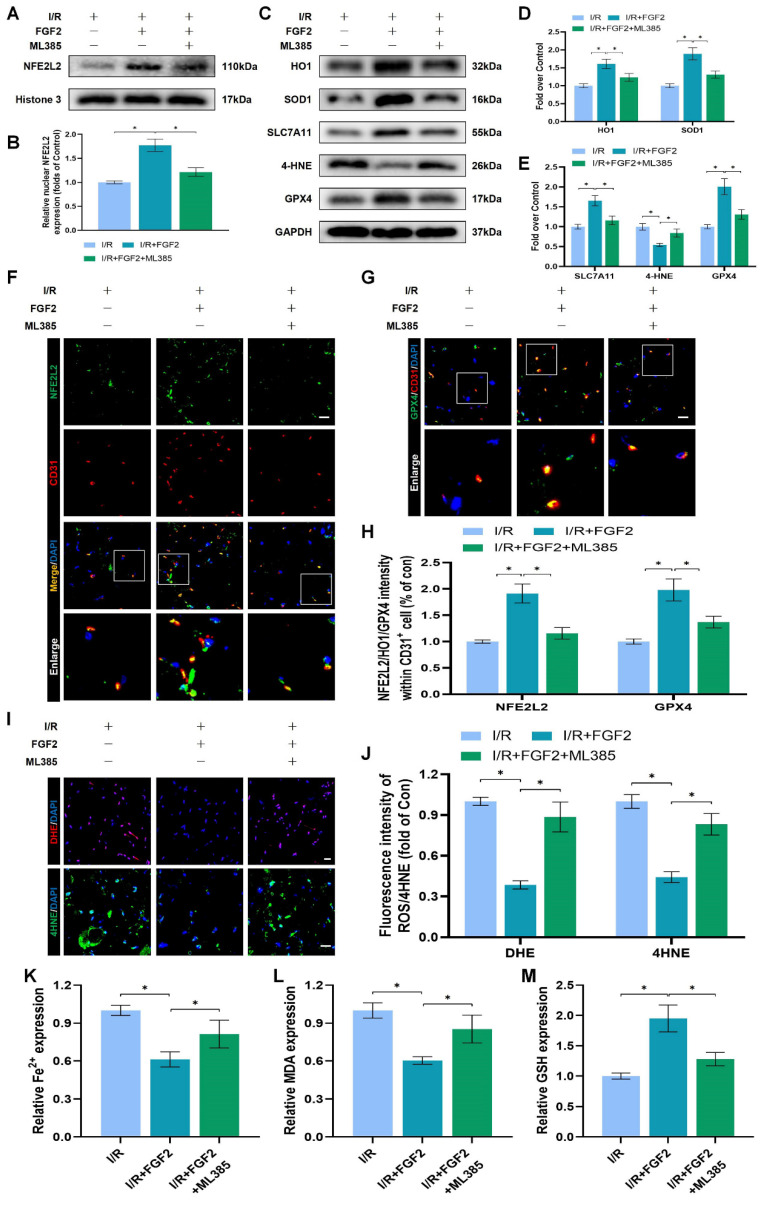
Through increased NFE2L2 activity, FGF2 mitigates oxidative stress and ferroptosis. (**A**) WB examination of skeletal muscle samples. (**B**) Quantification of protein level of NFE2L2 from (A) with normalized to Histone 3 band density. (**C**) WB assay of skeletal muscle tissues reveals the presence of HO-1, SOD1, SLC7A11, 4-HNE, and GPX4. (**D**) Quantification of the GAPDH band density-normalized HO-1 and SOD1 protein levels from (C). SLC7A11, 4-HNE, and GPX4 protein levels from (C) are quantified in **(E)**, normalised to GAPDH band density. (**F**) Micrographs of skeletal muscle slices with NFE2L2 and CD31, a marker for endothelial cells, immunostained; DAPI labelling outlined nuclei. Scale bars: 100 μm. (**G**) Micrographs of skeletal muscle slices with GPX4 and CD31 antibodies; DAPI labelling showed nuclei. Scale bars: 100 μm (**H**) Quantification of cells that express NFE2L2, GPX4, and CD31; ratios of co-expressing cells to all CD31-positive cells are shown. (**I**) Micrographs of skeletal muscle slices with nuclei identified by DAPI labelling and DHE and 4-HNE staining. Scale bars: 100 μm. **(J)** Quantification of the intensity of DHE and 4-HNE fluorescence from (I). (**K**) The concentration of Fe^2+^ is displayed as a histogram. (**L**) Content of MDA was displayed as a histogram. (**M**) Content of GSH was displayed as a histogram. Data are provided as mean ± SD (n = 3-5 per group). Significance: ^*^P < 0.05.

The above experimental results revealed that FGF2 has a protective effect in anti-OS and anti-ferroptosis. NFE2L2 is a classic transcription factor related to anti-oxidant response. Numerous investigations have shown that NFE2L2 protects against I/R damage by transcriptionally activating key genes involved in OS and ferroptosis signalling, which has led to its recent identification as a possible anti-ferroptosis agent 25, 26. We first evaluated NFE2L2 expression to see if FGF2 affects NFE2L2. Following FGF2 therapy, the WB analysis revealed that nuclear NFE2L2 protein expression increased in the skeletal muscle tissue (Fig. 5A, B), demonstrating FGF2's important influence on NFE2L2 activity.

After I/R injury, the nuclear NFE2L2 protein levels was then noticeably reduced after we added ML385, a novel NFE2L2 inhibitor (Fig. S2F, G). In comparison to the FGF2-only group, co-administration of ML385 and FGF2 dramatically reduced nuclear NFE2L2 protein expression following I/R damage (Fig. 5A, B). Then, we carried out an experiment to compare three cohorts, I/R, I/R+FGF2, as well as I/R+FGF2/ML385, to learn more about the main molecular defence mechanism of FGF2 against OS and ferroptosis *in vivo*.

Further WB analysis revealed that the administration of ML385 dramatically reduced the protein expression of HO-1, SOD1, SLC7A11, and GPX4, pointing to ML385's potential role in reversing FGF2's ability to defend against OS and ferroptosis following I/R injury (Fig. 5C-E). Immunofluorescence staining also consistently demonstrated that ML385 eliminated the fluorescence signal strength of NEF2L2 on the vascular wall in skeletal muscle following I/R injury (Fig. 5F, H). Application of ML385 also reduced the rise in GPX4 fluorescence signal strength brought on by FGF2 (Fig. 5G, H). Additionally, silencing of NFE2L2 minimized the fluorescence signal strength of 4-HNE in skeletal muscle tissue (Fig. 5I, J). The decreased level of ROS by FGF2 was also abrogated by ML385 administration (Fig. 5I, J). We found that reduced MDA and iron ion levels and increased GSH levels induced by FGF2 were also counteracted by ML385 administration (Fig. 5K-M).

To demonstrate whether FGF2 reduces I/R damage by modifying NFE2L2-dependent anti-OS and anti-ferroptosis effect, we observed a substantial reduction in limb blood perfusion within the I/R+FGF2/ML385 cohort relative to the I/R+FGF2 cohort (Fig. S2A, B). After ML385 treatment, the reduced wet-to-dry weight ratio was also increased (Fig. S2C). Furthermore, the decreased injured muscle fiber after FGF2 intervention was also reversed by ML385 (Fig. S2D, E). Taken together, these findings reveal that FGF2 inhibits OS and suppresses ferroptosis by activating NFE2L2.

### FGF2 inhibits OS and ferroptosis and ameliorates limb I/R injury via the KLF2-NFE2L2 axis

**Figure 6:**
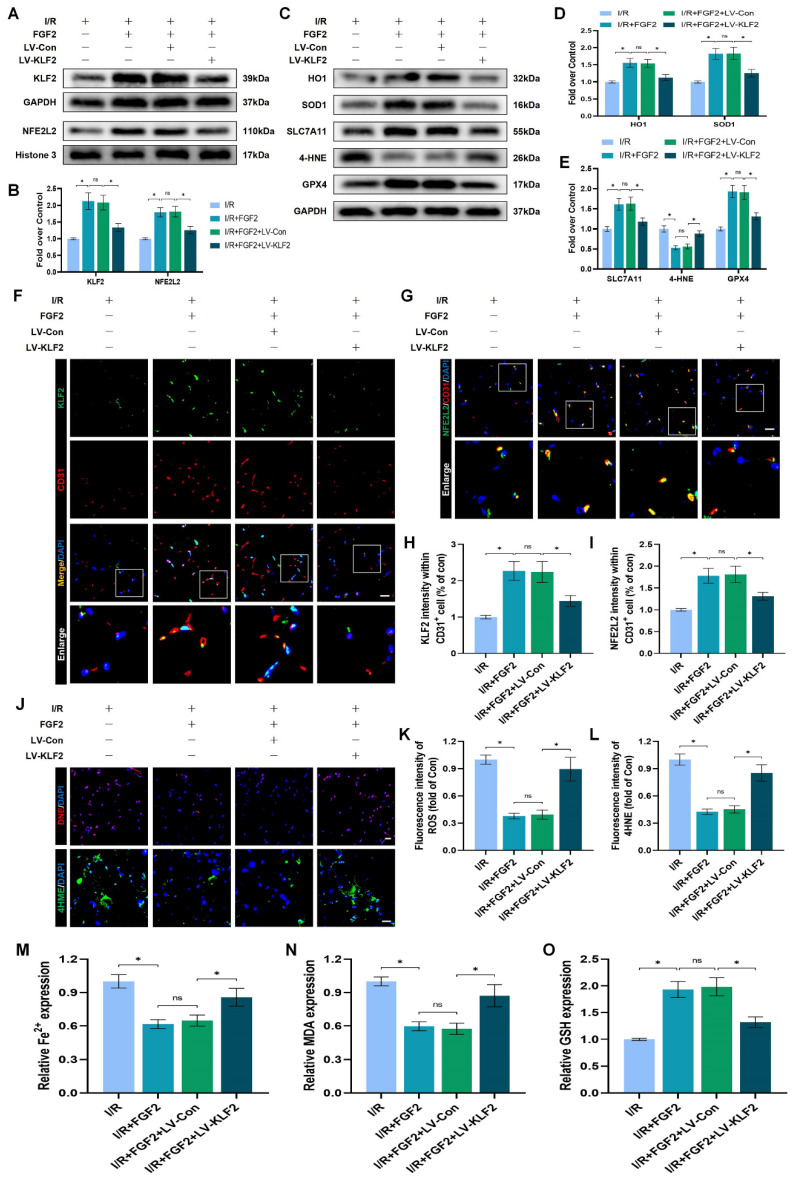
FGF2 reduces oxidative stress and ferroptosis via KLF2-NFE2L2 pathway *in vivo*. (**A**) Skeletal muscle specimens were used for the KLF2 and NFE2L2 WB assay. (**B**) Protein quantification of KLF2 and NFE2L2 from (A), normalised to GAPDH band density and histone 3 band density, respectively. (**C**) Skeletal muscle specimens showing HO-1, SOD1, SLC7A11, 4-HNE, and GPX4 on a WB assay. (**D**) Quantification of the GAPDH band density-normalized HO-1 and SOD1 protein levels from (C). (**E**) Quantification of protein level of SLC7A11, 4-HNE and GPX4 from (C) with normalized to GAPDH band density. (**F**) DAPI staining shows the nuclei in these micrographs of skeletal muscle slices that have been immunostained for KLF2 and CD31. Scale bars: 100 μm. (**G**) Micrographs of skeletal muscle slices with NFE2L2 and CD31 immunostaining; DAPI labelling shows nuclei. Scale bars: 100 μm. (**H**) Quantification of cells that co-express KLF2 and CD31; proportions of co-expressing cells to all CD31-positive cells are shown. (**I**) Quantification of NFE2L2 and CD31 double-positive cells, the ratio of co-expressing cells to all CD31-positive cells is shown. (**J**) Micrographs of skeletal muscle slices with nuclei identified by DAPI labelling and DHE and 4-HNE staining. Scale bars: 100 μm. Based on the immunofluorescence data in (J), the ROS level was quantified in **(K)**. Based on the immunofluorescence results in (J), 4-HNE fluorescence intensity was quantified in **(L)**. (**M**) Content of Fe^2+^ was plotted as a histogram. (**N**) The concentration of MDA is shown as a histogram. (**O**) The concentration of GSH is shown as a histogram. Data are provided as mean ± SD (n = 3-5 per group). Significance: ns, not significant; ^*^P < 0.05.

The experimental results indicated that FGF2 protected vascular ECs by increasing NFE2L2-mediated anti-OS and anti-ferroptosis functions. KLF2 is a classical vascular endothelial protective factor and has been shown to protect vascular endothelium in various vascular diseases 16, 48. Current evidence suggests that KLF2 can promote the expression of NFE2L2 by regulating the binding sites on NFE2L2 18, 24. Therefore, we speculated whether FGF2 activates NFE2L2 by regulating KLF2 activity. WB assay revealed that FGF2 administration remarkably augmented the levels of KLF2 in I/R-induced skeletal muscle tissue, suggesting that FGF2 can facilitate the activity of KLF2 (Fig. 6A, B). We next silenced the expression of KLF2 by using lentivirus. KLF2 levels were decreased in the KLF2-lentivirus group, according to Western blot analysis (Fig. S3F, G), suggesting that KLF2 expression was successfully suppressed by KLF2-lentivirus transfection. The I/R+FGF2 and I/R+FGF2/LV-Con groups showed no appreciable differences (Fig. 6A, B). Furthermore, we found that the knockdown of KLF2 reversed the protein expression levels of NFE2L2 by FGF2 intervention. The I/R+FGF2 and I/R+FGF2/LV-Con cohorts showed no appreciable differences (Fig. 6A, B). Subsequently, we explored whether FGF2-induced KLF2 activation can inhibit OS and suppress ferroptosis. WB assay revealed that silencing KLF2 could downregulate the protein expression degrees regarding HO1, SOD1, SLC7A11 and GPX4. Additionally, downregulated 4-HNE protein expression induced by FGF2 was augmented after KLF2-lentivirus (Fig. 6C-E). Immunofluorescence staining also consistently indicated that the FGF2-induced enhancement of the KLF2 fluorescence signal strength on the vascular wall of skeletal muscle was countered by KLF2-gene silencing (Fig. 6F, H). Moreover, KLF2-lentivirus treatment reduced NFE2L2 signal levels on the vascular wall in skeletal muscle were obstructed by the application of KLF2-lentivirus (Fig. 6G, I). Our results also revealed that KLF2-lentivirus could increase the fluorescence intensities of 4-HNE in skeletal muscle tissue (Fig. 6J, L). We also found that the decreased level of ROS by FGF2 was abrogated by KLF2-lentivirus (Fig. 6J, K). The reduced MDA and iron ion levels and increased GSH levels induced by FGF2 intervention were inhibited by KLF2-lentivirus (Fig. 6M-O).

We looked more closely at the potential pharmacological advantages of FGF2 following KLF2 lentivirus transfection in order to determine whether increasing KLF2 transcriptional activity could lessen limb injury. KLF2-gene silencing eliminated FGF2's protective effects on blood perfusion in limbs (Fig. S3A, B) and muscle edema (Fig. S3C). We found that the application of KLF2-lentivirus also remarkably enhanced the injured muscle fiber (Fig. S3D, E). In summary, these results reveal that FGF2 facilitates NFE2L2-mediated anti-oxidant response and anti-ferroptosis by enhancing KLF2 transcriptional activity.

### FGF2 inhibits OS and ferroptosis via the KLF2-NFE2L2 signaling axis *in vitro*

**Figure 7:**
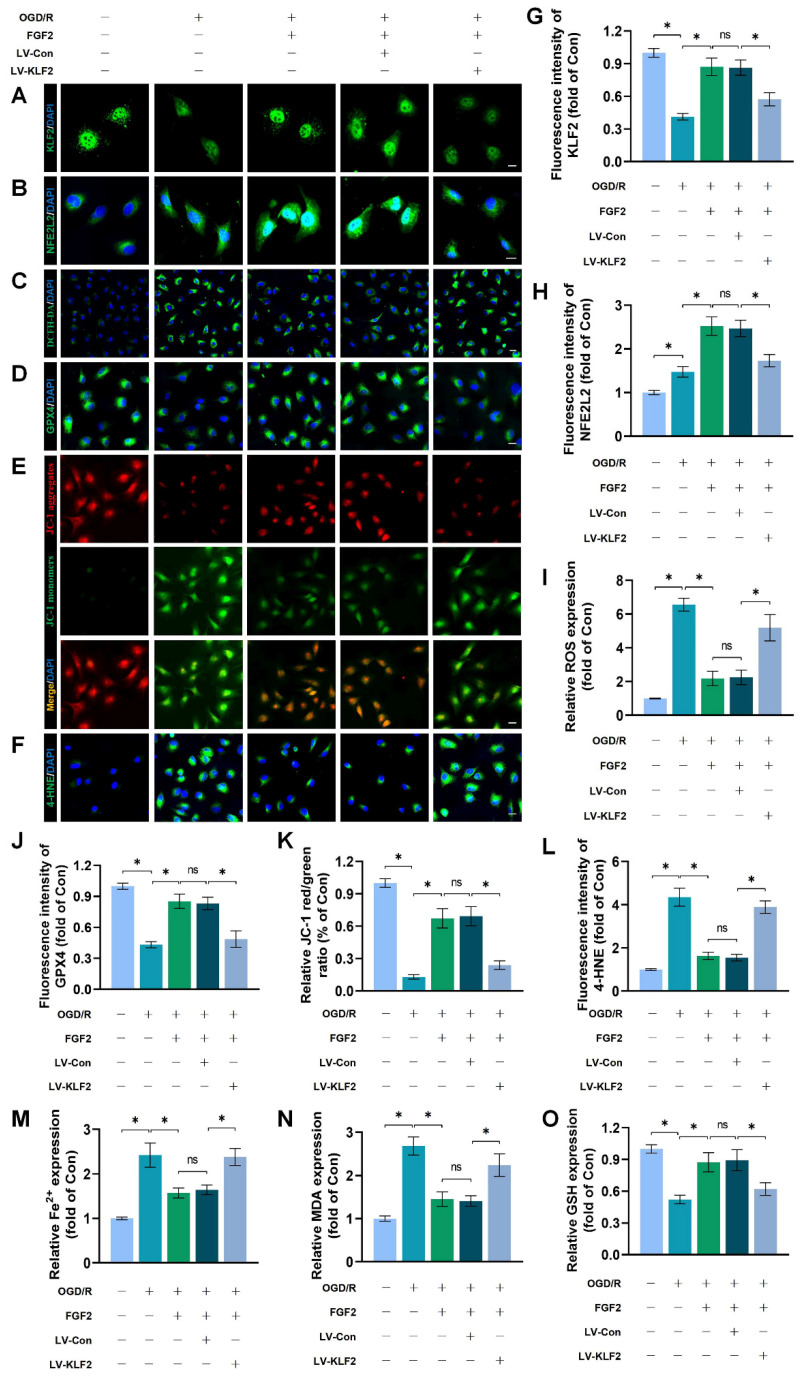
FGF2 reduces oxidative stress and ferroptosis through KLF2-NFE2L2 axis *in vitro*. (**A**) KLF2-labeled HUVECs slices show DAPI-stained nuclei that can be distinguished. Scale bars: 25 μm. (**B**) Images of HUVECs sections stained with NFE2L2, nuclei were recognized by DAPI staining. Scale bars: 25 μm. (**C**) Images of HUVECs sections stained with DCFH-DA, nuclei were recognized by DAPI staining. Scale bars: 25 μm. (**D**) Images of HUVECs sections stained with GPX4, nuclei were recognized by DAPI staining. Scale bars: 25 μm. (**E**) Images of HUVECs sections stained with JC-1. Scale bars: 25 μm. (**F**) Using DAPI staining, 4-HNE-stained HUVECs sections show different nuclei. Scale bars: 25 μm. Immunofluorescence data from (A) are calculated in **(G)**, which shows the mean optical density of KLF2. Analysing immunofluorescence data from (B) reveals that NFE2L2 has an average optical density **(H)**. (**I**) Quantification of immunofluorescence data from (C) displaying the level of ROS. The mean optical density of GPX4 is shown in **(J)** by measuring the immunofluorescence data from (D). (**K**) Quantification of immunofluorescence data from (E) displaying the level of ferroptosis. The average optical density of 4-HNE is shown in **(L)** based on the analysis of immunofluorescence data from (F). (**M**) Content of Fe^2+^ was plotted as a histogram. (**N**) A histogram showing the content of MDA. (**O**) GSH content as shown by a histogram. Data are shown as the means ± SD (n = 3 per group). Significance: ns, not significant; ^*^P < 0.05.

To explore the promising role and underpinning molecular mechanisms of FGF2 inhibiting OS and ferroptosis via the KLF2-NFE2L2 signaling axis, we performed *in vitro* experiments on HUVECs. Our immunofluorescence findings exhibited that reduced KLF2 minimized the fluorescence intensities of KLF2 and NFE2L2 and no remarkable difference was found between OGD/R+FGF2 and OGD/R+FGF2/LV-Con group (Fig. 7A-B, G-H). These results demonstrated that the lentivirus of KLF2 downregulated KLF2 and NFE2L2 activities in HUVECs. Moreover, the knockdown of KLF2 also remarkably augmented the levels of ROS in HUVECs measured by DCFH-DA staining (Fig. 7C, I). Additionally, the fluorescence signal strength of GPX4 on HUVECs was reduced and the downregulation of KLF2 abolished the protective effects of FGF2 on anti-ferroptosis in OGD/R-treated HUVECs (Fig. 7D, J). No significant differences were observed between the OGD/R+FGF2 and OGD/R+FGF2/LV-Con groups. The fluorescence signal strength of 4-HNE on HUVECs was remarkably enhanced, which consistently suggested the protection of FGF2 on anti-ferroptosis in OGD/R-treated HUVECs was reversed by KLF2-lentivirus (Fig. 7F, L). There were no appreciable differences between the OGD/R+FGF2 and OGD/R+FGF2/LV-Con groups. MMP was evaluated by JC-1 detection and JC-1 red/green ratio correlates negatively with levels of ferroptosis. The fluorescence signal strength of the JC-1 red/green ratio was markedly decreased by using KLF2-lentivirus compared to the LV-Con group. There were no appreciable differences between the OGD/R+FGF2 or OGD/R+FGF2/LV-Con groups (Fig. 7E, K).

We found that decreased MDA and iron ion levels and augmented GSH induced by FGF2 were also inhibited by lentivirus KLF2 (Fig. 7M-O) in OGD/R-treated HUVECs. Taken together, these findings revealed that FGF2 facilitated NFE2L2-mediated anti-oxidant response and anti-ferroptosis by enhancing KLF2 transcriptional activity.

### FGF2 activates KLF2 via the AMPK-HDAC5 signaling pathway

**Figure 8:**
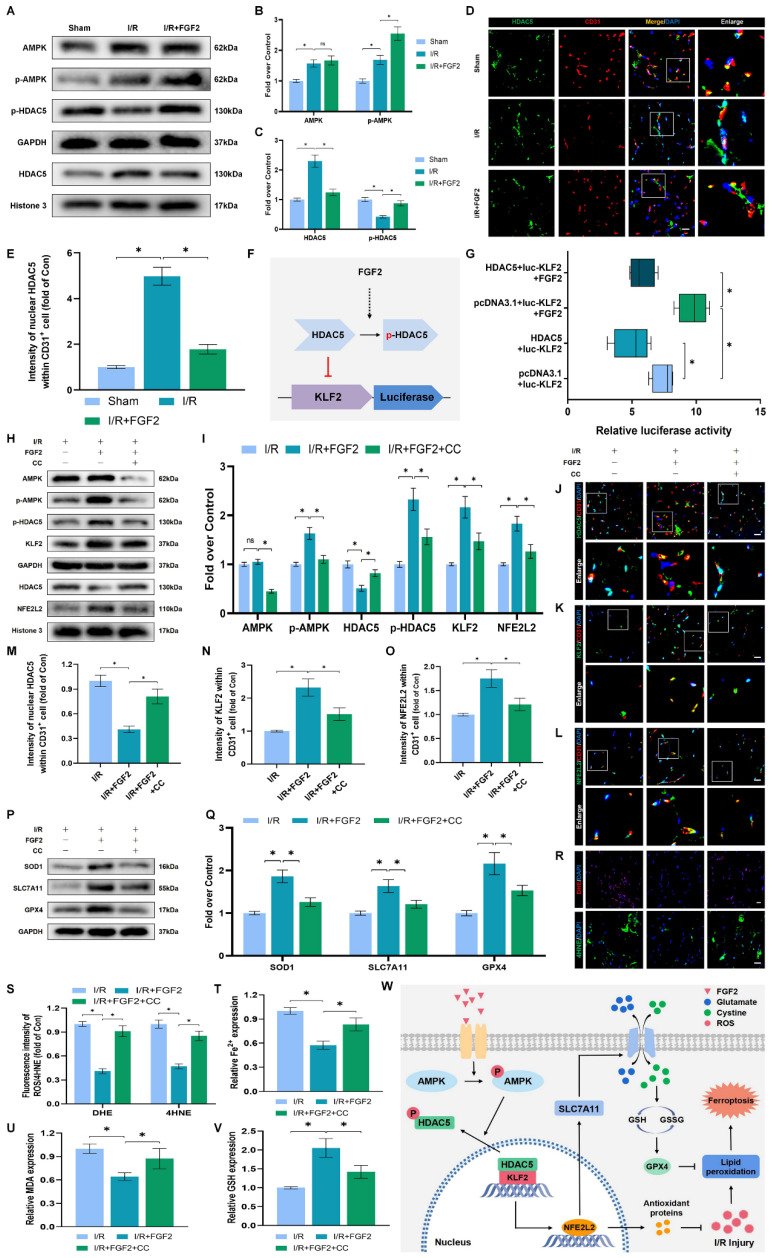
FGF2 activates KLF2 through the AMPK-HDAC5 pathway. (**A**) Skeletal muscle tissues' WB assay for AMPK, p-AMPK, p-HDAC5, and HDAC5. AMPK and p-AMPK protein levels from (A) are quantified in **(B)**, normalised to GAPDH band density. HDAC5 protein level from (A) is evaluated in **(C)**, normalised in respect to Histone 3 band density, coupled with p-HDAC5 level from (A), normalised in relation to GAPDH band density. **(D)** Using DAPI labelling, skeletal muscle slices labelled with HDAC5 and the EC marker CD31 reveal distinct nuclei. Scale bars indicate 100 μm. Nuclear HDAC5 and CD31 double-positive cells computation **(E)**. (**F**) Images from a dual luciferase reporter gene test. (**G**) Relative luciferase activity was plotted as a box plots. (**H**) Skeletal muscle tissues' WB assay for AMPK, p-AMPK, p-HDAC5, KLF2, HDAC5, and NFE2L2. (**I**) Quantification of protein level of AMPK, p-AMPK, p-HDAC5, KLF2 from (H) with normalized to GAPDH band density, HDAC5 and NFE2L2 protein levels from (H) with normalized to Histone 3 band density. (**J-L**) Images of skeletal muscle slices with DAPI-stained nuclei that have had HDAC5, KLF2, NFE2L2, and EC marker CD31 stained. Scale bars: 100 μm. (**M-O**) Quantification of nuclear HDAC5, KLF2, NFE2L2 and CD31 double-positive cells, the percentages of double positive cells versus total CD31 positive cells are indicated. (**P**) Skeletal muscle tissues' WB assay for SOD1, SLC7A11, and GPX4. SOD1, SLC7A11, and GPX4 protein levels are evaluated in **(Q)** and normalised based on GAPDH band density. (**R**) Photographs of skeletal muscle slices with distinct nuclei shown by DAPI staining and DHE and 4-HNE staining. Scale bars: 100 μm. Immunofluorescence data from (R) are measured in **(S)**, which reveals the average optical density of 4-HNE and the degree of ROS. (**T**) Content of Fe^2+^ was plotted as a histogram. (**U**) A histogram showing the content of MDA. (**V**) A histogram showing the GSH content. (**W**) Schematic representation of proposed molecular mechanisms highlighting the roles of AMPK-HDAC5 signaling, KLF2, NFE2L2, oxidative stress, ferroptosis in the pathophysiology of I/R-induced microvascular injury in extremities. Data are presented as the mean ± SD (n = 3-5 per group). Significance: ns, not significant; ^*^P < 0.05.

We conducted more research to understand the molecular mechanisms behind FGF2. The HDAC family is a class of deubiquitinating enzymes 39. Past studies have revealed that HDAC5 regulates KLF2 and the phosphorylation of HDAC5 may further enhance HDAC5 nuclear translocation 39. We investigated whether the regulation of KLF2 by FGF2 is achieved by modulating HDAC5. Firstly, we examined the content of HDAC5. Our WB assay revealed that FGF2 can increase HDAC5 phosphorylation (Fig. 8A, C), and immunofluorescence labelling confirmed that FGF2 can decrease nuclear HDAC5's fluorescence intensity on the skeletal muscle vascular wall, hence boosting HDAC5 phosphorylation (Fig. 8D, E). We next performed the dual-luciferase assay to analyze whether FGF2 enhances the transcriptional activity of KLF2. HDAC5 was overexpressed in 293T cells by transfecting pcDNA3.1-HDAC5. Dual-luciferase reporter gene analysis later revealed that FGF2 augmented ranscriptional activity of KLF2 by enhancing luciferase activity driven by the KLF2 promotor, while overexpression of HDAC5 inhibited the transcriptional activity of KLF2 (Fig. 8F, G). AMPK is a serine/threonine protein kinase complex. Studies have revealed that FGF2 can activate AMPK to exert diverse biological functions 38. AMPK induces HDAC5 cytoplasmic/nuclear translocation under stress conditions 40. Our outcomes revealed that FGF2 activated p-AMPK in the cytoplasm, but there were no discernible variations in AMPK expression between the I/R and FGF2 cohorts (Fig. 8A, B). Overall, these findings demonstrate that the AMPK-HDAC5 signalling pathway is activated by FGF2.

We investigated the pharmacodynamics of CC, a selective AMPK inhibitor with respect to the AMPK-HDAC5 signalling pathway and limb preservation to determine if the AMPK-HDAC5 signalling pathway precipitates FGF2-mediated KLF2 activation. Our results exhibited that CC significantly reduced p-AMPK protein expression levels within the I/R group (Fig. S4F, G). More importantly, WB assay revealed that CC obstructed FGF2-mediated phosphorylation of AMPK and HDAC5 (Fig. 8H, I). Intriguingly, there were no noticeable differences in AMPK expression between the FGF2 and FGF2+CC cohorts. We also found that increased level of KLF2 and NFE2L2 mediated by FGF2 was also inhibited by CC administration (Fig. 8H, I). By increasing the strength of nuclear HDAC5's fluorescence signal on the vascular wall of skeletal muscle, immunofluorescence labelling confirmed that CC reduced HDAC5 phosphorylation (Fig. 8J, M).

Reduced fluorescence intensities of KLF2 and NFE2L2 were seen on the vascular wall after AMPK pathway inhibition (Fig. 8K-L, N-O). These results demonstrate that FGF2 activates the AMPK-HDAC5 pathway and enhances KLF2 and NFE2L2 nuclear translocation. Interestingly, when CC was administered, the positive benefits were reversed. Then, we looked into whether the AMPK-HDAC5 pathway controls the antioxidant response and anti-ferroptosis actions induced by FGF2. After AMPK pathway inhibition, the expression of the SOD1, SLC7A11, and GPX4 were significantly downregulated, according to a WB experiment (Fig. 8P, Q). In addition, immunofluorescence staining displayed that CC remarkably enhanced the fluorescence signal strength of 4-HNE in skeletal muscle tissue (Fig. 8R, S). Our results indicated that the decreased level of ROS induced by FGF2 was also reversed by CC (Fig. 8R, S). To analyze other indicators of ferroptosis, we found that reduced MDA and iron ion levels and increased GSH levels induced by FGF2 were also counteracted by CC (Fig. 8T-V). Finally, we explored whether enhancing KLF2 transcriptional activity by FGF2 through AMPK-HDAC5 may ameliorate limb injury. Our findings show that AMPK activity inhibition counteracts FGF2's protective effects on limb perfusion (Fig. S4A, B) and muscle edema (Fig. S4C), while CC administration counteracts FGF2's protective effects on muscle fibre damage (Fig. S4D, E). These observations collectively show that FGF2 promotes KLF2-NFE2L2-mediated anti-oxidant response and anti-ferroptosis by activating the AMPK-HDAC5 signaling axis.

## Discussion

The common vascular injury known as acute lower-limb ischemia is characterised by a high incidence rate and a dismal prognosis. Organ function resuscitation continues to be accomplished only by restoring blood flow to ischemic tissue 49. Reperfusion therapy paradoxically has certain intrinsic hazards and the potential to amplify damage 50. Microvascular endothelial cell damage is visible in the early stages of I/R injury due to its direct contact with injury factors in the reperfusion blood flow 51. Considering that microvascular injury occurs in the early stage of reperfusion and further aggravates the tissue damage, ameliorates microvascular damage in the early stage of injury can largely avoid the biological consequences of I/R injury. Furthermore, the principal reason of aberrant microvascular function in I/R injury is still EC death 52. A growing body of research points to OS as a key component advancing microvascular damage after I/R injury 5.

Ferroptosis can induce EC death, which is different from other types of programmed death 30, 31. Previous studies have reported that FGF2 minimizes infarct size and ameliorates microvascular dysfunction 34, 35. In our research, we found that FGF2 exerts promising anti-oxidative functions as well as alleviates EC death within lower-limb I/R injury by suppressing ferroptosis-driven cell death.

A major contributing element to lower limb I/R damage is OS, according to growing body of evidence 53, 54. It is widely believed that OS is triggered by an imbalance in the production of oxygen radicals and anti-oxidants, which impairs the functions of normal cells. The reinstitution of blood flow to ischemic tissue is followed by excessive ROS formation, which further disrupts cellular macromolecules and induces cell death 53, 55, 56. Numerous ROS are a major cause of EC damage, according to previous research 57-59. Ferroptosis is induced by free peroxidation of lipids and subsequent rupture of plasma membranes 60. Ferroptosis is associated with a variety of physiological and pathological processes. It has been discovered that ferroptosis is closely implicated in I/R injury in the heart, brain, liver and kidney. Multiple studies have revealed that ferroptosis induces ischemic injury, which may lead to the initiation of I/R injury 11, 13. Given the existing studies showing that FGF2 exerts various biological functions such as pro-angiogenesis, wound healing, tissue regeneration and anti-oxidant activity 30, 31, we speculated that applying FGF2 in acute lower-limb I/R model could alleviate the injury by obstructing OS signals and attenuating ferroptosis. Current research shows that FGF2 augments the expression of NFELE2 and alleviates I/R injury 61, 62. NFELE2 is a transcription factor recognized as the primary defensive mechanism against OS that coordinates the activation of cytoprotective genes 63. The kelch-like ECH-associated protein 1 (KEAP1) tightly regulates intracellular concentration of NFE2L2 64, 65. More and more data suggests that OS promotes the oxidation of KEAP1's active cysteine residues, releasing NFE2L2 into the nucleus 66-68. To modulate the production of HO-1 and SOD1, the conglomerated NFE2L2 complex sequence-specifically connects to AREs in the promoter regions of target genes 69, 70. NFE2L2 is an essential transcriptional regulator of ferroptosis-mediated cellular death 71. Ferroptosis can be induced by extrinsic or intrinsic pathways 72. The intrinsic pathway is activated by blocking intracellular anti-oxidant enzymes such as GPX4 13. GPX4 is a key anti-oxidant enzyme in the regulation of ferroptosis. The presence of GSH and selenium modulates the expression and function of NFELE2 in ferroptosis, which disrupts membrane lipid hydroperoxides to non-toxic lipid alcohols 73, 74. The extrinsic pathway is activated by blocking cell membrane transporters including the cystine/glutamate transporter (also referred to as system xc-). System xc- reaches cells and transports oxidized cysteine, which modulates glutathione formation via glutamate cysteine ligase (GCL) and impairs the expression and function of GPX4 13. The xc- system comprises two subunits: SLC7A11 (also known as xCT) and SLC3A2.

Likewise, genetic depletion of SLC7A11 or GPX4 triggers lipid peroxidation and ferroptosis 75, 76. NFE2L2 also positively regulates SLC7A11 expression and activity 77. Emerging evidence has revealed that ferroptosis is inhibited by the activation of NFE2L2/HO-1-related pathways 71, 78. Furthermore, NFELE2 ameliorates oxidative damage during ferroptosis by activating several cytoprotective genes involved in iron and GSH metabolism and ROS detoxification enzymes 79. In our study, we used Fer-1 (a ferroptosis inhibitor) and Erastin (a ferroptosis inducer) and found that FGF2 administration efficaciously suppressed the levels of ferroptosis, comparable to Fer-1 treated. Besides, the previously suppressed EC death levels induced by FGF2 were augmented after Erastin application, indicating that Erastin could remarkably reverse the protective impact of FGF2 upon attenuating ferroptosis. These prove that ferroptosis is engaged in I/R injury of the lower limbs and FGF2 protects against I/R by attenuating ferroptosis in ECs. Our results indicated that FGF2 suppressed EC death by obstructing ROS and lipid peroxides, promoting the expression of anti-oxidant genes and attenuating ferroptosis genes. In addition, ML385 (an NFELE2 inhibitor) was employed to antagonize FGF2-mediated activation of NFELE2 signaling and found that anti-OS and anti-ferroptosis effects induced by FGF2 were considerably reversed. In conclusion, FGF2 exerts promising anti-OS and anti-ferroptosis effects via the NFELE2 signaling axis.

We further investigated the upstream mechanism of NFE2L2 to analyse the crucial functions and intrinsic molecular mechanisms of FGF2 in reducing lower-limb I/R damage. KLF2 is one of the families of transcription factors containing conserved zinc-finger domains mainly expressed by ECs and regulates endothelial functions 22, 80. Considerable evidence indicates that KLF2 exerts remarkable antithrombotic, anti-inflammatory and anti-OS effects in I/R-associated diseases 20, 21, 81. KLF2 coordinates the expression of numerous genes that regulate the ferroptosis process and suppress ROS formation by activating NFE2L2 82. In particular, the literature has reported that the KLF2-induced gene spectrum such as NQO1, HO-1, glutamate cysteine ligase modification subunit (GCLM) and catalase (CAT) that targets NFE2L2 18. Therefore, we speculated that KFL2 activation could obstruct the stimulation of OS and suppress ferroptosis-driven cell death by enhancing the expression of NFE2L2. In this research, we evaluated the promising functions of KLF2. Our data revealed that activated KLF2 significantly augmented the expression levels of NFE2L2 and restricted OS and suppressed ferroptosis in the FGF2 group. However, we found that increased anti-OS and anti-ferroptosis induced by FGF2 were reversed by applying lentivirus KLF2. More importantly, our results reveal that FGF2 obstructs OS, inhibits ferroptosis and alleviates lower-limb I/R injury via activating KLF2.

We investigated how FGF2 alters KLF2 activity since it offers a solid foundation for prospective therapeutic uses. This was done in light of FGF2's encouraging therapeutic potential. The activation of the AMPK pathway inhibits the development of I/R-related illnesses, according to earlier research 36, 37, 83. Recent studies have suggested that AMPK phosphorylation may be induced by FGF2 38. In addition, activation of the AMPK pathway might encourage HDAC5 to move from the nucleus to the cytoplasm 40. HDAC5 is a suppressor of angiogenesis. Urbich and co-workers recently reported that silencing HDAC5 exhibits a unique pro-angiogenic effect that facilitates the migration of ECs 41.

Furthermore, the phosphorylation of HDAC5 could facilitate the activation of KLF2 41, 42. Our findings indicated that FGF2 could augment the phosphorylation of AMPK in acute lower-limb I/R injury. We revealed that FGF2 intervention could trigger the HDAC5-KLF2 signaling pathway and enhance NFE2L2 activity. More specifically, our data indicated that FGF2 intervention inhibited the activation of OS and alleviated ferroptosis-dependent microvascular cell death. We used CC (an AMPK blocker) to inhibit FGF2-mediated signaling pathway activation. We observed that the previously suppressed ferroptosis was offset and the enhanced anti-oxidant response was decreased. In summary, we revealed that FGF2 inhibits EC death and attenuates I/R damage by augmenting the nuclear translocation of NFE2L2 in the lower limb via the AMPK-HDAC5-KLF2 signalling pathway.

Interestingly, the remarkable therapeutic effect of FGF2 suggests the promising clinical translation approach. However, before this avenue is applied clinically, some clinical challenges remain to be addressed: 1) Intraperitoneal administration of FGF2 may lead to rapid diffusion in the body that reduces the effective concentration of the ischemic limb and may cause adverse side effects. Intravascular administration may be an ideal factor for ischemic or I/R-associated diseases. 2) The underlying molecular mechanism of lower-limb I/R injury is complex and interactive. Other pathological mechanisms must be evaluated for the appropriate drug combination 52. 3) In addition to the improvement of microcirculation, FGF2 may also directly protect skeletal muscle. Some new experiments, such as employing conditional KLF2-knockout mice to clarify the functions of FGF2 in EC and/or skeletal muscle under I/R damage situations.

## Conclusions

Our research revealed that FGF2 dramatically ameliorate microvascular damage, reduces hypoperfusion, tissue edema and skeletal muscle fiber injury in limbs following I/R. In our research, we discovered that FGF2 stimulates the AMPK-HDAC5 signalling pathway, which increases KLF2 activity. Importantly, I/R-induced microvascular damage is reduced and limb viability is increased thanks to FGF2-induced KLF2 activation, which also supports the NFE2L2-mediated inhibition of OS and ferroptosis in limb ECs (Fig. 8W). Overall, our research suggests FGF2 as a potentially effective therapeutic target for microvascular treatment in limb I/R injury.

## Supplementary Material

Supplementary figures.

## Abbreviations

4-HNE: 4-Hydroxynonenalal
AMPK: AMP-activated protein kinase
ARE: antioxidant response element
CC: compound C
DEGs: differentially expressed genes
DHE: dihydroethidium
DMEM: Dulbecco's Modification of Eagle's Medium
ECs: endothelial cells
FBS: Fetal Bovine Serum
Fer-1: ferrostatin-1
FGF2: fibroblast growth factor 2
FGFR: fibroblast growth factor 2 receptor
GPX4: glutathione peroxidase 4
GSH: glutathione
HDAC5: histone deacetylase 5
HO-1: heme oxygenase-1
HUVECs: human umbilical vein endothelial cells
I/R: ischemia/reperfusion
KLF2: kruppel-like factor 2
LDI: laser doppler imaging
MDA: malondialdehyde
MMP: mitochondrial membrane potential
NFE2L2: nuclear factor E2-related factor 2
NQO1: NAD(P)H:quinone oxidoreductase 1
OGD/R: Oxygen-glucose deprivation/re-oxygenation
OS: oxidative stress
PBS: phosphate-buffered saline
PFA: paraformaldehyde
ROS: reactive oxygen species
SLC7A11: solute carrier family 7, member 11
SOD1: superoxide dismutase 1
