# Supplementary material for: FGF2 Alleviates Microvascular Ischemia-Reperfusion Injury by KLF2-mediated Ferroptosis Inhibition and Antioxidant Responses
Source: Int J Biol Sci. 2023 Aug 21;19(13):4340–59. doi: 10.7150/ijbs.85692 (PMC10496511; doi:10.7150/ijbs.85692)
Supplement: Supplementary file 1 — Supplementary figures. [file ijbsv19p4340s1.pdf]

Supplementary Information

for

FGF2 Attenuates Microvascular Ischemia-Reperfusion Injury by KLF2-mediated Ferroptosis Inhibition And Antioxidant Responses

Fanfeng Chen *et. al*

Figure S1

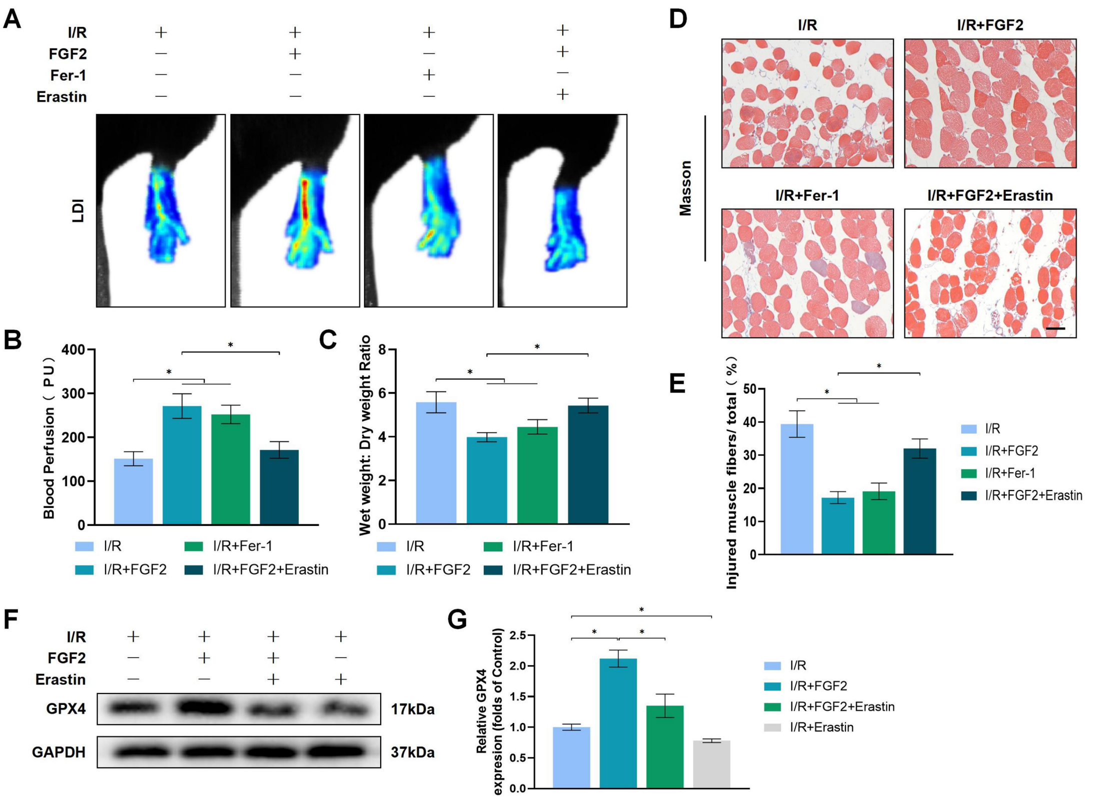

Figure S1. FGF2 attenuates limb I/R injury by inhibiting ferroptosis. (**A**) Blood perfusion of hind limbs were detected by LDI. (**B**) Signal intensity of blood flow was plotted as a histogram. (**C**) Wet weight to dry weight ratio. (**D**) Masson staining of the transverse sections of skeletal muscle. Scale bar, 100 μm. (**E**) The degree of skeletal muscle fiber injury was assessed by the percentage of injured fibers. (**F**) Western blots for GPX4 in skeletal muscle tissues. (**G**) Quantification of protein level of GPX4 from (F) with normalized with respect to GAPDH band density. Data are displayed as the means ± SD (n = 3-4 per group). Significance: $^{*}P < 0.05$.

Figure S2

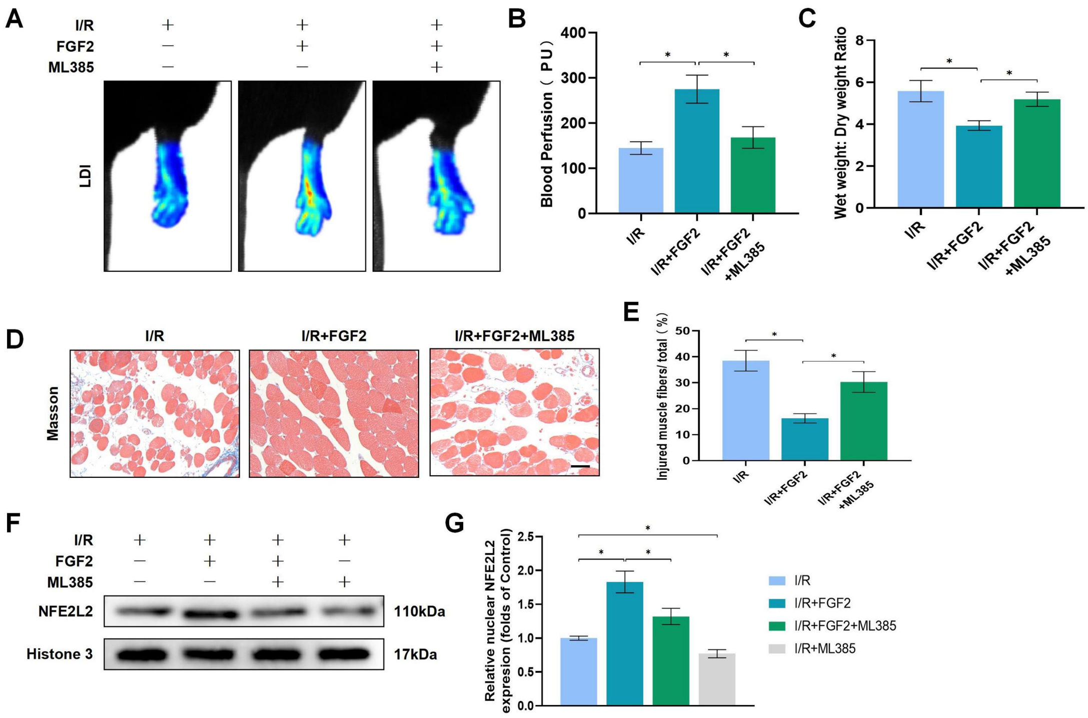

Figure S2. FGF2 attenuates limb I/R injury by activiting NFE2L2. (**A**) Blood perfusion of hind limbs were detected by LDI. (**B**) Signal intensity of blood flow was plotted as a histogram. (**C**) Wet weight to dry weight ratio. (**D**) Masson staining of the transverse sections of skeletal muscle. Scale bar, 100 μm. (**E**) The degree of skeletal muscle fiber injury was assessed by the percentage of injured fibers. (**F**) Western blots for nuclear NFE2L2 in skeletal muscle tissues. (**G**) Quantification of protein level of NFE2L2 from (F) with normalized with respect to Histone 3 band density. Data are displayed as the means ± SD (n = 3-4 per group). Significance: $^{*}P < 0.05$.

Figure S3

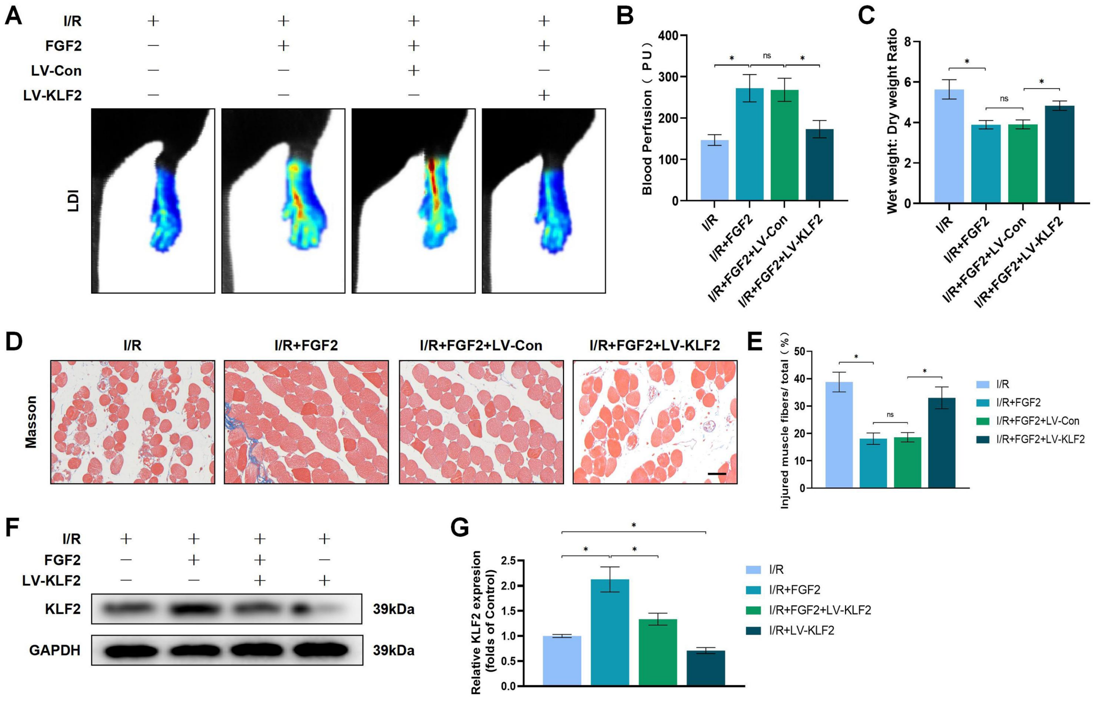

Figure S3. FGF2 attenuates limb I/R injury via the KLF2-NFE2L2 axis. (**A**) Blood perfusion of hind limbs were detected by LDI. (**B**) Signal intensity of blood flow was plotted as a histogram. (**C**) Wet weight to dry weight ratio. (**D**) Masson staining of the transverse sections of skeletal muscle. Scale bar, 100 μm. (**E**) The degree of skeletal muscle fiber injury was assessed by the percentage of injured fibers. (**F**) Western blots for KLF2 in skeletal muscle tissues. (**G**) Quantification of protein level of KLF2 from (F) with normalized with respect to GAPDH band density. Data are displayed as the means ± SD (n = 3-4 per group). Significance: ns, non-significant; $^{*}P < 0.05$.

Figure S4

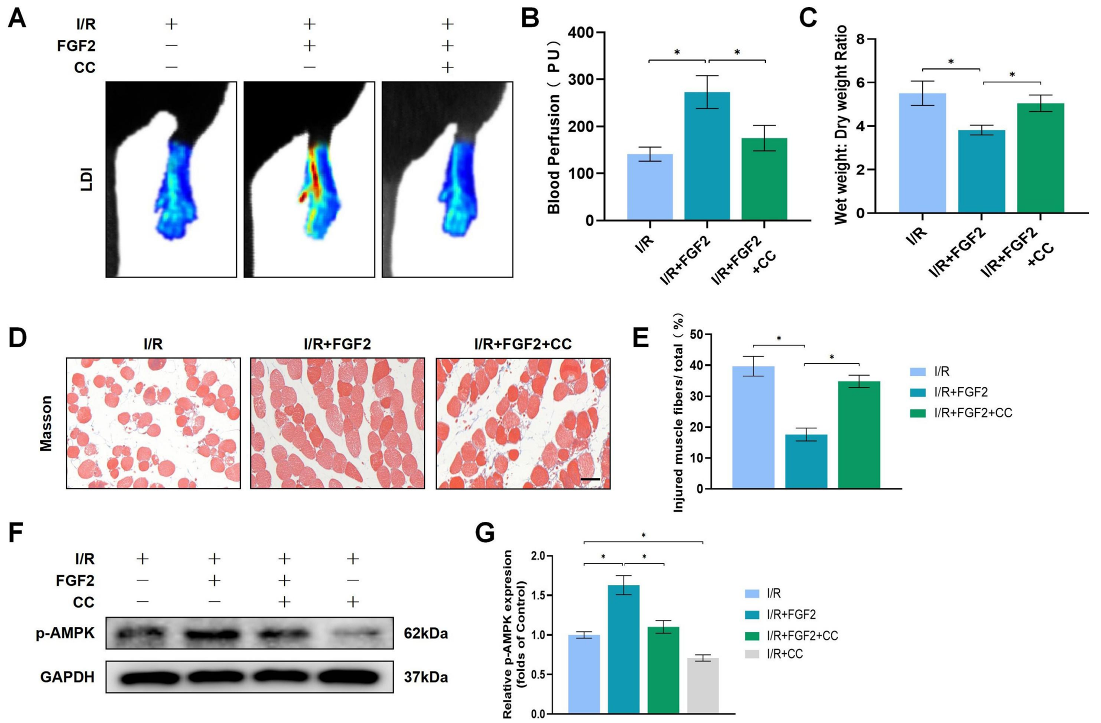

Figure S4. FGF2 attenuates limb I/R injury via the AMPK signaling pathway. (**A**) Blood perfusion of hind limbs were detected by LDI. (**B**) Signal intensity of blood flow was plotted as a histogram. (**C**) Wet weight to dry weight ratio. (**D**) Masson staining of the transverse sections of skeletal muscle. Scale bar, 100 μm. (**E**) The degree of skeletal muscle fiber injury was assessed by the percentage of injured fibers. (**F**) Western blots for nuclear p-AMPK in skeletal muscle tissues. (**G**) Quantification of protein level of p-AMPK from (F) with normalized with respect to GAPDH band density. Data are displayed as the means ± SD (n = 3-4 per group). Significance: $^{*}P < 0.05$.
